# Multifidus muscle retracted and preserved in resection of malignant bone tumor in the sacral ala

**DOI:** 10.1093/jscr/rjab439

**Published:** 2021-10-12

**Authors:** Akio Sakamoto, Bungo Otsuki, Takashi Noguchi, Shuichi Matsuda

**Affiliations:** Department of Orthopaedic Surgery, Graduate School of Medicine, Kyoto University, Kyoto, Japan; Department of Orthopaedic Surgery, Graduate School of Medicine, Kyoto University, Kyoto, Japan; Department of Orthopaedic Surgery, Graduate School of Medicine, Kyoto University, Kyoto, Japan; Department of Orthopaedic Surgery, Graduate School of Medicine, Kyoto University, Kyoto, Japan

## Abstract

Resection of malignant tumors in the posterior pelvis requires multidirectional approaches for the resection and the subsequent spine-pelvic fixation. The multifidus muscle can be scarified during the operation. This is a case report of a 44-year-old male with a secondary chondrosarcoma arising from an osteochondroma in the sacral ala. Recurrence occurred 11 months after the initial operation, and the resected tissue from the recurrence was diagnosed as a chondrosarcoma. In both operations, the multifidus muscle was elevated from its distal attachment to provide an adequate view of the tumor resection and insertion of spine-pelvic instrumentation. An adequate view by elevation of the multifidus muscle is useful for a safe operation. A preserved multifidus muscle covering the instrumentation may reduce the risk of infection. The elevation and preservation of the multifidus muscle is an easy and simple method that contributes to successful resection of a malignant tumor of the pelvis.

## INTRODUCTION

Resection of a malignant bone tumor in the pelvis is challenging because the structure of the pelvis is complex, especially in resection of a pelvic tumor of the posterior ilium or sacral ala [1]. The resection of the pelvic tumor sometimes requires fixation with instrumentation between the spine and the pelvis. In the tumor resection, multidirectional approaches using anterior, lateral and posterior approaches are necessary for an adequate surgical field, and the posterior approach is necessary for subsequently inserting instrumentation. To expose the posterior aspect of the vertebrae and posterior ilium the multifidus muscle can become an obstacle to the clear view of the surgical field, and scarification of the multifidus muscle may occur in this case. In this article, a method for the preservation of the multifidus muscle is introduced using a representative patient with secondary chondrosarcoma arising from osteochondroma in the sacral ala.

## CASE REPORT

**Figure 1:**
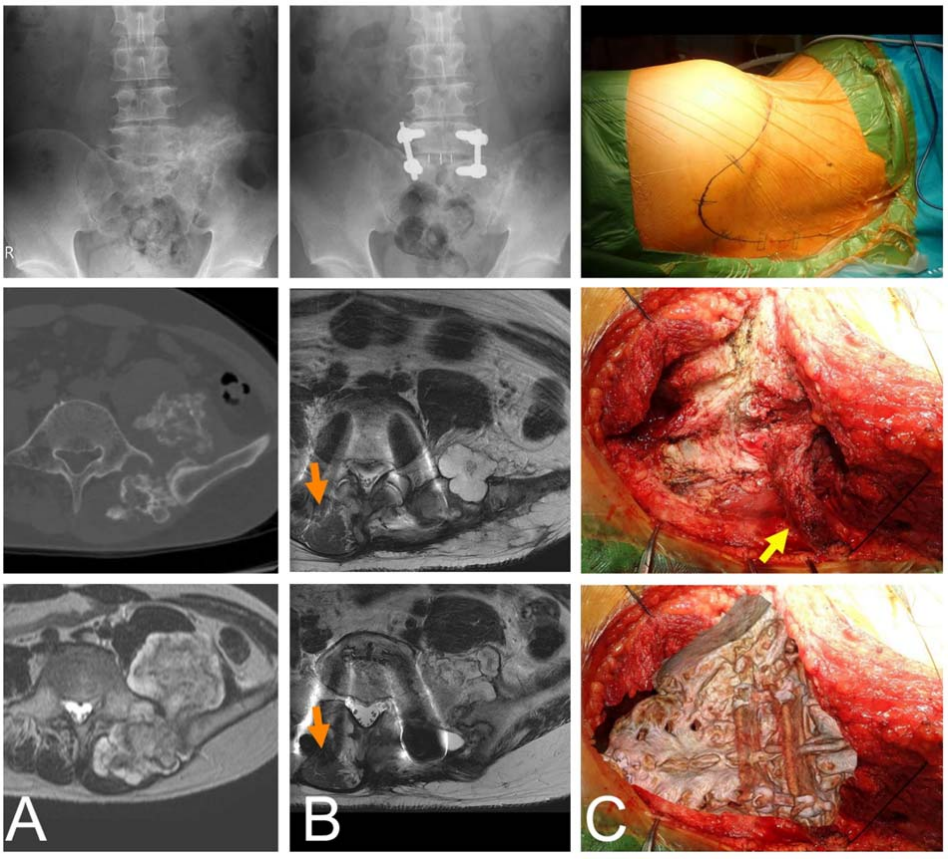
Chondrosarcoma in the sacrum of a 44-year-old male. A plain radiograph (**A**, top) and CT (A, middle) shows an osseous lesion in the sacrum. The osseous lesion is covered by a cartilaginous lesion with a high signal T2 weighted image (A, bottom). L5 and S1 were fused with screws in the initial operation (**B**, top). Eleven months after the initial operation cartilaginous nodules appear as a recurrent lesion at L5 (B, middle) and S1 (B, bottom). The once elevated distal multifidus muscle on the non-tumor side was morphologically almost normal (orange arrows), but the distal multifidus muscle on the tumor side was still thin. The patient was in the right-side bottom decubitus position. The skin marker shows the convex skin incision at the distal sacrum posteriorly (**C**, top). The sacrum is viewed by retraction of the multifidus muscle (yellow arrow) (C, middle). The image of the sacrum is overlapped on the C-middle picture to show the anatomical location (C, bottom).

A 44-year-old male initially presented with pain in his lower extremity. He visited a nearby hospital that referred him to our institute where a working diagnosis of a bone tumor was made. The plain radiograph shows an osseous lesion on the left posterior pelvis. Computed tomography (CT) depicted an osseous lesion arising from the left sacral ala. The osseous lesion was covered by a layer with intermediate signal intensity on the T1 weighted image and high signal intensity on the T2 weighted image from magnetic resonance imaging. The diagnosis from the image was an osteochondroma, which was covered by a cartilaginous cap. The maximum size of the tumor was ~6 cm and the thickness of the cartilaginous cap was <2 cm (Fig. 1A). The histology of the needle biopsy of the cartilaginous cap showed a proliferation of chondroid tissue without atypia, suggesting an osteochondroma.

On diagnosis of the osteochondroma, a resection was performed. With the patient in the lateral position, a skin incision was made along the iliac crest to the contralateral paravertebral muscle, with a distal convex shape at the sacrum. The distal multifidus muscle was detached from the lamina of the sacral and lumber vertebrae. The posterior ilium and vertebrae were exposed, and the tumor was resected. Following resection, a vertebral fusion between L5 and S1 was performed using pedicle screws (Fig. 1B). No complications associated with the operation appeared. The pathological diagnosis of the resected material was osteochondroma. The patient was able to walk without a crutch though dull back pain existed 6 months after the surgery with the degree of the pain getting worse. Image analysis 11 months after the initial operation revealed new cartilaginous nodules. The distal multifidus muscle on the tumor side was still thin, but the distal multifidus muscle on the non-tumor side was morphologically almost normal **(**Fig. 1B**)**.

**Figure 2:**
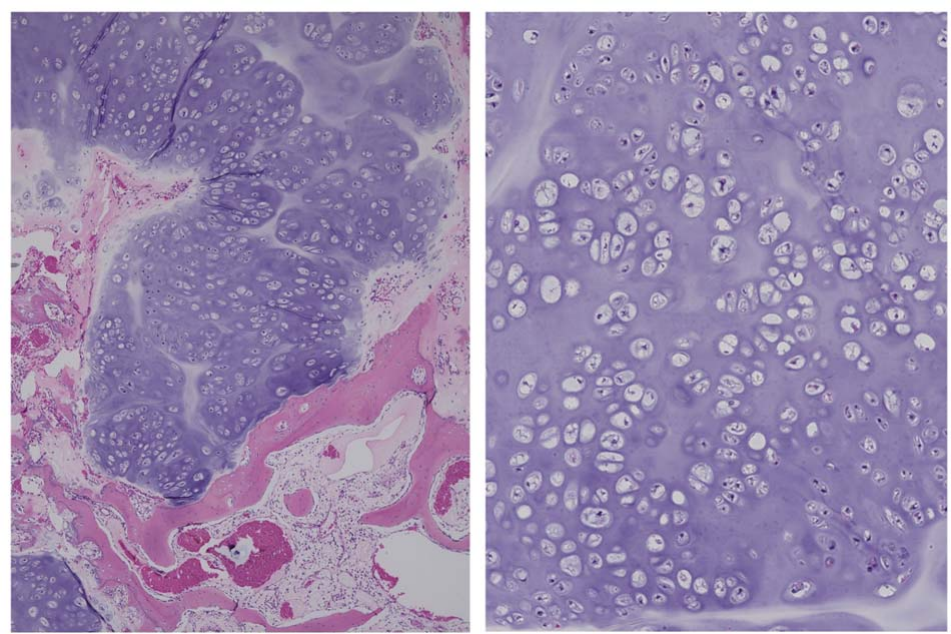
The same case as in Fig. 1, diagnosed as a chondrosarcoma. The resected recurrent tumor has a nodular structure with cartilaginous matrix. A permeative pattern is observed (left). The magnified view shows dense chondrocytes (right).

A needle biopsy of the new tissue revealed no atypical cartilaginous tissue. However, taking into consideration the clinical course, chondrosarcoma was diagnosed. Surgery was performed with almost the same incision, but the incision was extended to the distal sacrum (Fig. 1C). The multifidus muscle was removed from the distal sacrum in the same way as in the primary operation (Fig. 1C). The tumor compressed the L5 nerve root and was resected in several blocks. The pathological diagnosis of the resected material was a chondrosarcoma arising from the osteochondroma (Fig. 2). The pain was relieved postoperatively.

## DISCUSSION

The merit of this preservation method of the multifidus muscle is that it provides good exposure of the anatomically complex posterior pelvis and spine during tumor resection and subsequent instrumentation. The preserved multifidus muscle also covers the instrumentation at wound closure.

The multifidus muscles are paraspinal muscles that are essential for carrying physiological loads and which provide stability to the lumbar spine [2]. Using this new method, the preservation of the multifidus muscle was confirmed at the second operation. However, it was not conclusive whether the preserved multifidus muscle was functional because muscle detachment from the spinous processes and muscle stripping can lead to muscle atrophy after spinal surgery [3, 4]. Generally, prolonged muscle retraction in lengthy surgeries is also a factor leading to muscle atrophy [4]. Muscle atrophy is usually more prominent at the caudal adjacent levels than the cranial adjacent levels [3]. Though the method requires detachment of the muscle from the distal lumbar vertebrae and the sacrum, the method avoids prolonged retraction that could damage the muscle in these regions. Electromyography might be useful to confirm the function of the multifidus muscle.

Pelvic surgery for malignant bone tumors has a high wound complication rate, with 39% resulting in infection [5]. Muscle-based flaps are known to provide an adequate tissue mass for eliminating dead space; dead space increases the risk of infection after hemipelvectomy [6]. The preserved multifidus muscle does not have a large volume, but the preserved muscle can cover the instrumentation in the sacrum. This could reduce the infection rate following pelvic surgery for a malignant tumor.

In the current case, a diagnosis of secondary chondrosarcoma eventually was made, but the initial diagnosis was a benign osteochondroma. A cartilage cap thickness of >2 cm in osteochondroma is reported to distinguish a secondary chondrosarcoma from a benign osteochondroma [7, 8]. In the current case, the thickness was <2 cm in all parts. In a previous report, the cartilage cap of the secondary chondrosarcoma arising from the osteochondroma ranged from 0.5 cm to 15.0 cm [9, 10]. Thus, a thickness of the cartilage cap <2 cm is not an absolute factor to exclude chondrosarcoma. It has been reported that preoperative histology of a biopsy correctly predicted the final histological grade in only 27% of secondary chondrosarcoma cases occurring from osteochondroma [7]. This is a reminder of the difficulty in diagnosis of secondary chondrosarcoma.

The method preserving the multifidus muscle provides a good field of view in surgery for secondary chondrosarcoma airing from osteochondroma in the sacral ala and allows coverage of the bone and the instrumentation. Muscle coverage of the instrumentation may reduce the risk of infection.

## CONFLICT OF INTEREST STATEMENT

None declared.

## FUNDING

None.
